# VITILIGO: IS IT JUST A DERMATOLOGICAL DISORDER?

**DOI:** 10.4103/0019-5154.39745

**Published:** 2008

**Authors:** Harsarl T Pandve

**Affiliations:** *From Department of Community Medicine, Dr. DY Patil, Medical College, Pimpri, Pune - 411 018, India. E-mail: dr_harshalpandve@yahoo.co.in*

Vitiligo is a common, acquired, discoloration of the skin, characterized by well-circumscribed, ivory or chalky white macules which are flush to the skin surface.^1^ It affects 1% of the population worldwide.^2^ Both sexes are affected equally. There is a gradual loss of pigment melanin from the skin layers which results in white patches. These patches look ugly, especially in persons with dark complexion. Vitiligo can occur at any age. The condition does not cause any organic harm. The highest incidence of the condition has been recorded in Indians from the Indian subcontinent, followed by Mexico and Japan. The difference in its incidence may be due to a higher reporting of vitiligo in a population where an apparent color contrast and stigma attached to the condition may force them to seek early consultation.^1^

Kent and Al'Abadie found a high level of distress in people with vitiligo compared to the general population.^3^ Immediate recognition of an individual's difference from the norm is through their appearance. Not only can appearance indicate an underlying difference but it can be seen as a source of deviance in itself. As such vitiligo is hardly a disease of medical significance but there is more of a social stigma attached to it because of cosmetic reasons. It, however, brings about great psychological tension to the patient who is more embarrassed than the victim of any pain or discomfort. These patches gradually increase in size and cause lot of psychological stress in the patient. While vitiligo is a physical disorder, the patches can be mistaken for dirty marks, a social deviance. With its cause not widely known, the individual may be blamed for their lesions. Thus vitiligo is not just the lack of skin pigment, but affects the entire identity of a person. In India, it is called ‘safed dag’ or ‘kod’ and it carries much social stigma as compared to other developed nations. In many parts of India due lack of education and deeply rooted superstitions, vitiligo is considered as punishment of past sins. Young women face more social stigma and suffer more due to matrimonial problems.

A common response is to conceal lesions, which avoids the possibility of stigmatization, but can cause anxiety and preoccupation with the concealment.^4^ To label a person as deviant and unacceptable may influence their self-concept, behavior, cognition and psychological health.^5^ Social isolation leads to loneliness, negative affect and ill health, changes in processing of social information, anxiety and even criminality.^6^

People with vitiligo have to cope with more than their skin disease. There is a strong need for supporting these people. Some of the NGOs are working for the support of these people globally. They provide a common platform for such people to discuss their problems, to exchange views, to learn scientific facts about the disorder and above all to improve the self-image of the person. In India also, a few NGOs are working on this issue. Apart from this, popular media like Films are also making their contribution to this issue. Recently, the Marathi Film “Nital” (2006) was based on the life of a young woman suffering from vitiligo. It touches the crux of the issue of self-image and acceptance. The story reflects the confusion and contradiction surrounding the disease. Such efforts of social commitment by different sectors must be appreciated and encouraged.

A more realistic way of reducing social isolation in people with vitiligo is to support them in exploring and using effective coping strategies.
